# Supplementary material for: Small-scale fisheries catch more threatened elasmobranchs inside partially protected areas than in unprotected areas
Source: Nat Commun. 2022 Aug 9;13:4381. doi: 10.1038/s41467-022-32035-3 (PMC9363485; doi:10.1038/s41467-022-32035-3)
Supplement: Supplementary file 1 — Supplementary Information [file 41467_2022_32035_MOESM1_ESM.docx]

Supplementary Materials for

Small-scale fisheries catch more threatened elasmobranchs inside partially protected areas than in unprotected areas

Manfredi Di Lorenzo, Antonio Calò, Antonio Di Franco, Giacomo Milisenda, Giorgio Aglieri, Carlo Cattano, Marco Milazzo, Paolo Guidetti

Correspondence to: [manfredi.dilorenzo@libero.it](mailto:manfredi.dilorenzo@libero.it); [antonio.difranco@szn.it](mailto:antonio.difranco@szn.it)

*Predictors used to run the model*

Several types of variables were considered for assessing the protection on elasmobranch in the 11 locations: anthropogenic, bathymetric, geographical, temporal and environmental (Supplementary Table 1). Among the anthropogenic variables, Location (9 levels, Cap Roux and Cote Bleue MPAs were removed from the analyses as they do not have a partially protected zone with fishing restrictions) and protection (2 levels: PPAs and unprotected) were included to assess the effect of protection on BCPUE and NCPUE; a measure of cumulative human impact (including several human activities) extracted from ^1^ was included as it could affect elasmobranchs behaviour. Latitude and longitude coordinates were considered as geographic variables, since our study was carried out in a long spatial gradient in the Mediterranean Sea. As the study was carried out along 16 months (between June 2017 and October 2018), we included the variable ‘season’ including four levels: “Winter” (December-March), “Spring” (March-June), “Summer” (June-September), “Autumn” (September-December), to control for a potential temporal variability.

Depth is one of the bathymetric gradients known to affect elasmobranchs distribution ^2–4^. Fishers provided the approximate depth for each catch. Type of habitat was also included being an important factor determining the distribution of demersal elasmobranchs ^4,5^. Data for this variable were sourced from the European Marine Observation and Data Network (EMODNET, <http://www.emodnet.eu/>) and attributed to six habitat classes: coarse and mixed sediment, rock or other hard substrata, *Posidonia oceanica* meadows, sand, muddy sand, sandy mud (https://eunis.eea.europa.eu/).

Six environmental variables were used as predictors of elasmobranch catches in the Mediterranean Sea (Supplementary Table 1). The monthly mean of chlorophyll a (chl-a) concentration (mg/m3) was obtained and used as a proxy for primary productivity in the area of interest, since its variability can modify trophic conditions of the species’ habitat from oligotrophic to mesotrophic ^3,6^. Dissolved oxygen (DOX), phosphate (PHO) and nitrate (NIT) were added as predictors given their importance on primary production. They have also been found to be among the main variables affecting elasmobranch distribution ^7^. Sea surface salinity (SSS) and sea surface temperature (SST) were selected as they are recognized to influence habitat selection of elasmobranch species ^3,6,8^. All the environmental data (Chl a, SSS, SST, PHO, NIT, DOX and salinity) were extracted as the monthly mean and 0.042 degree, temporal and spatial resolution respectively, from the Copernicus website (<https://marine.copernicus.eu/>).

Multicollinearity tests showed correlations between some variables (Supplementary Table 7). Dissolved oxygen and nitrate variables showed a collinearity effect when associated with ‘pho’ and ‘chlorophyll a’ respectively, generating high variance inflation factor (VIF) values when we performed the test (Supplementary Table 3). Hence, we excluded the dox and nit variables from the set of candidate models to select the best model from the data. The most parsimonious models we selected are reported in Supplementary Table 8. Residual plots of the GAMLSS (Generalized additive models for location, scale and shape) models within the zero-adjusted Gamma (ZAGA) model are compared in Supplementary Figure 4. Again, there was no significant differences in the soaking time and mesh size between PPAs and UPAs (X^2^ = 3.1203, Df = 1, pvalue =0.008; X^2^ =1.8953, Df =1, pvalue =0.1686, respectively).

**
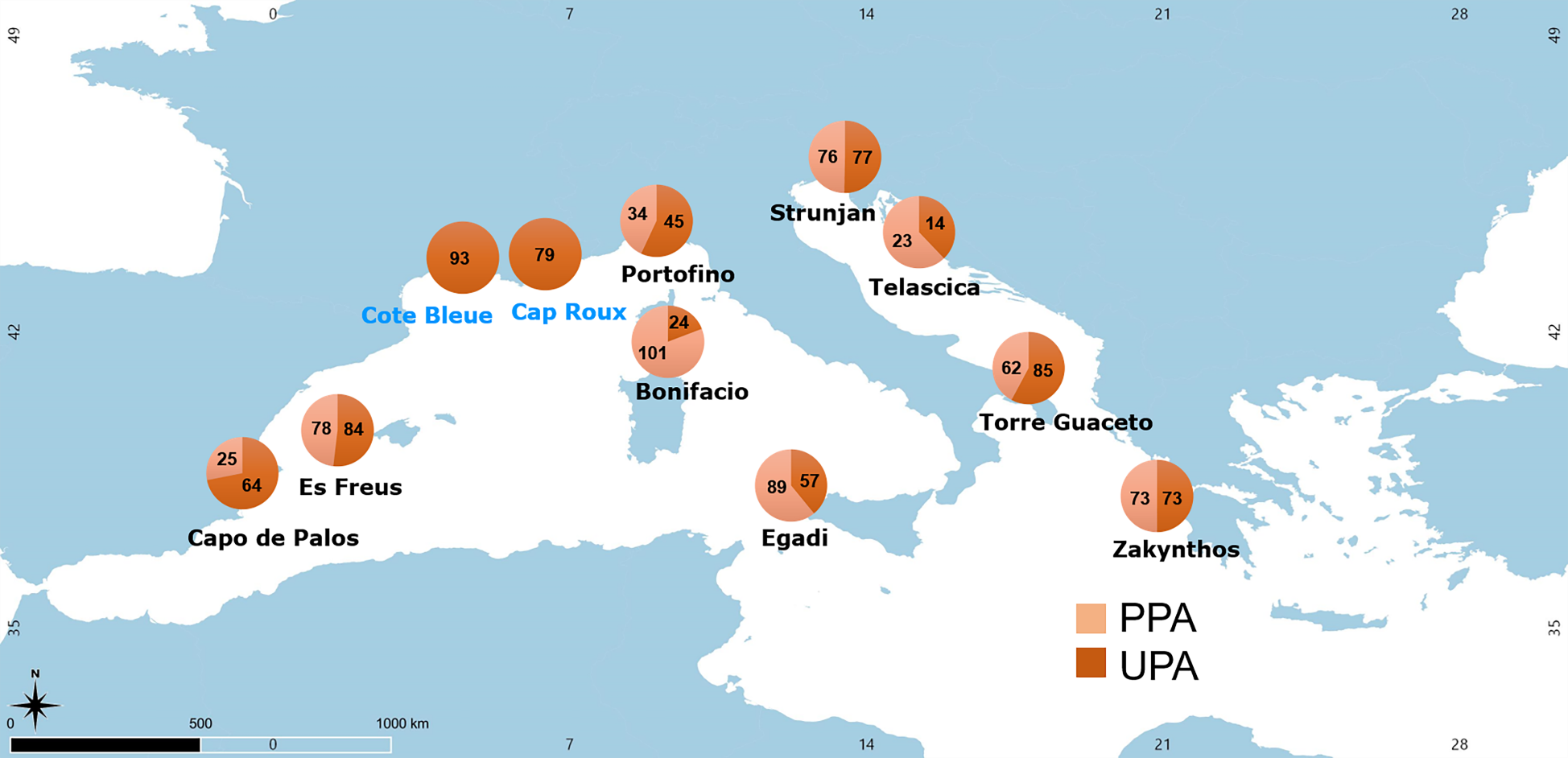
**

Supplementary Figure 1. **Number of fishing operations monitored in each location inside and outside the PPAs**. Numbers of fishing operations assessed in each location are indicated inside the pie charts. Names in black indicate multiple-use MPAs; blue ones indicate MPAs including only fully protected areas. Source data are provided as a Source Data file.

**
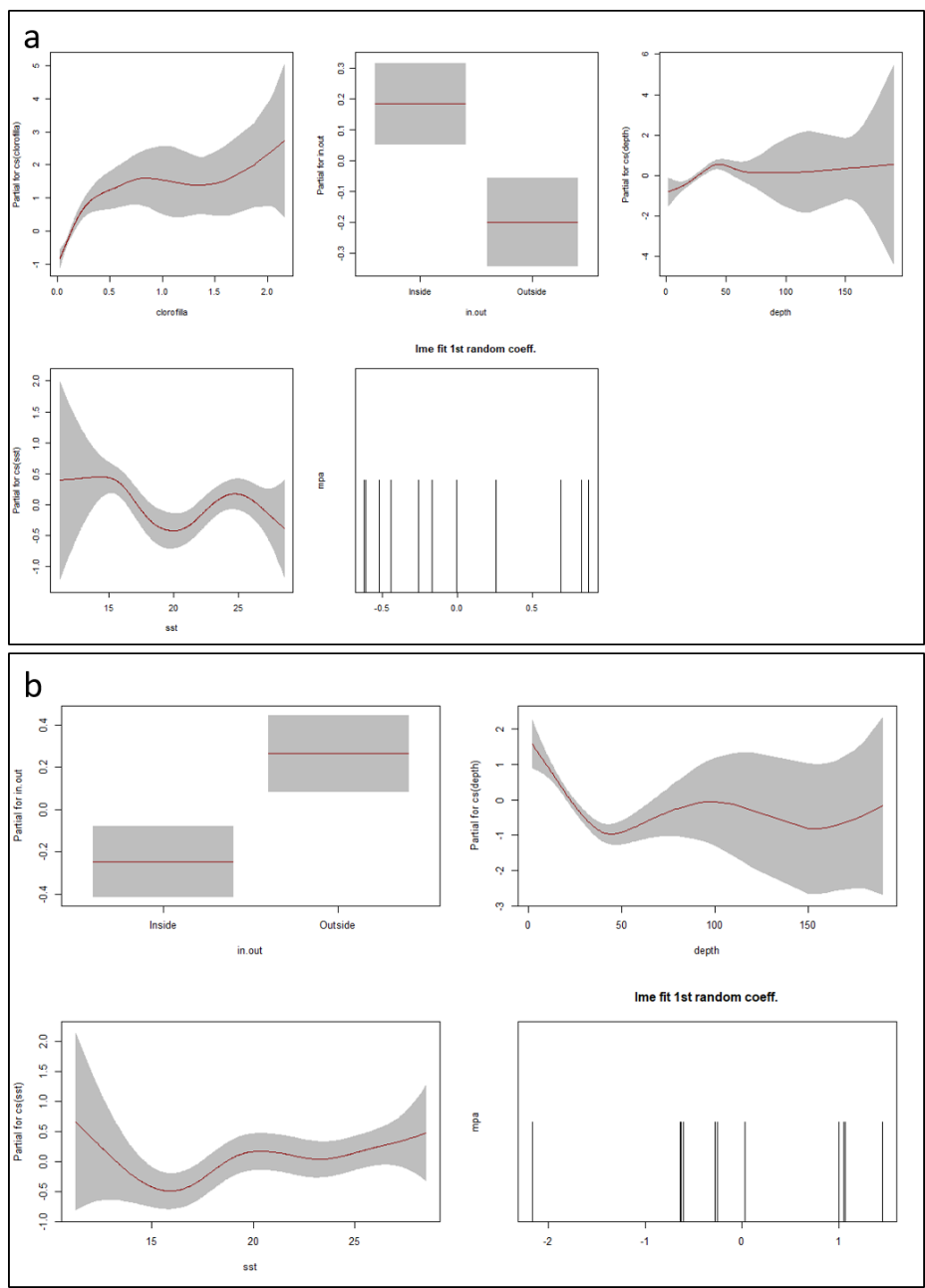
**

Supplementary Figure 2. **The effect of different variables on Biomass Caught per Unit Effort (BCPUE, kg /1000 net) of elasmobranchs, based on the GAMLSS model. a) counting part of the model, b) binomial part of the model**


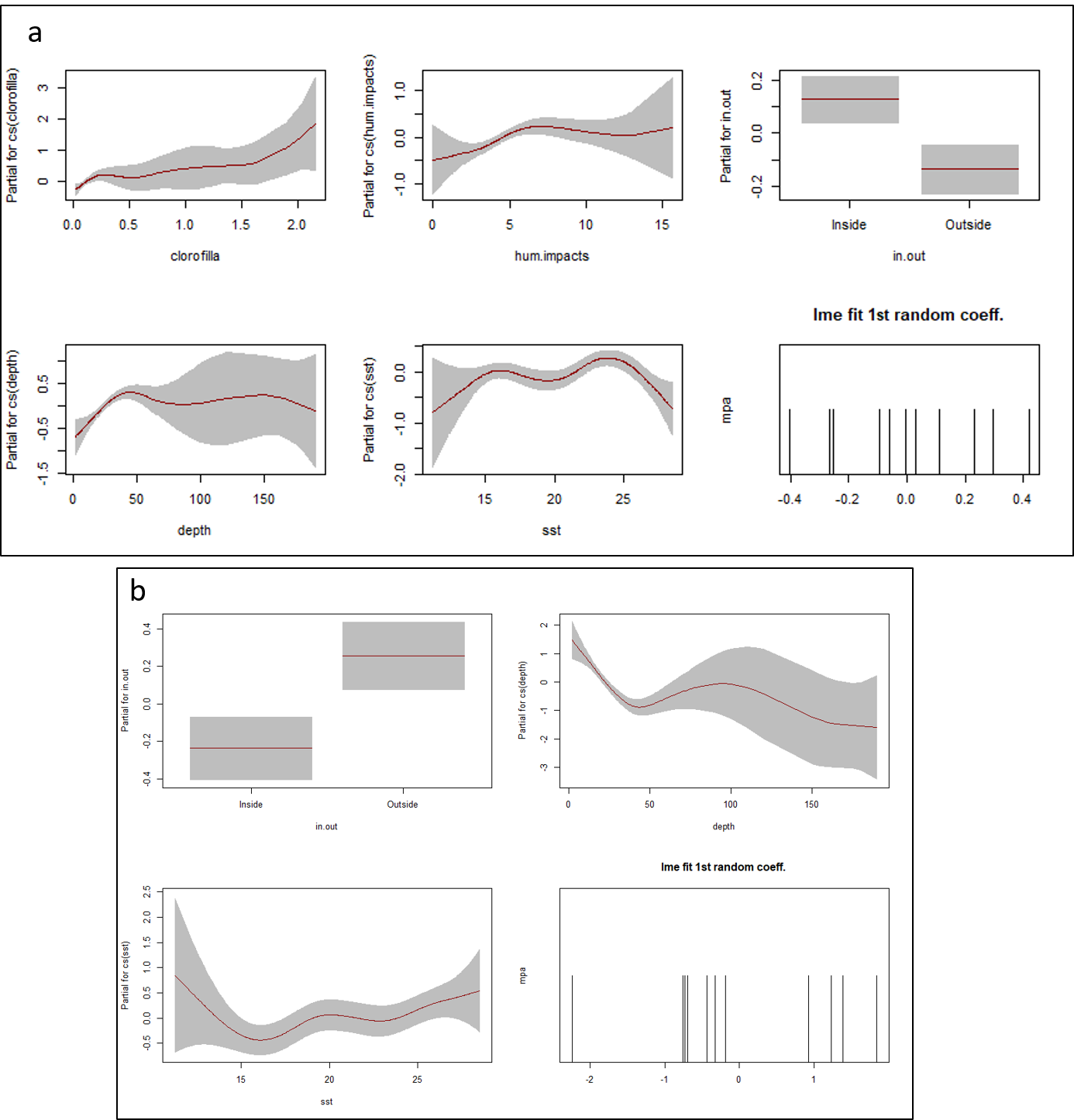


Supplementary Figure 3. **The effect of different variables on number of individuals Caught per Unit Effort (NCPUE, kg /1000 net) of elasmobranchs, based on the GAMLSS model. a) counting part of the model, b) binomial part of the model**


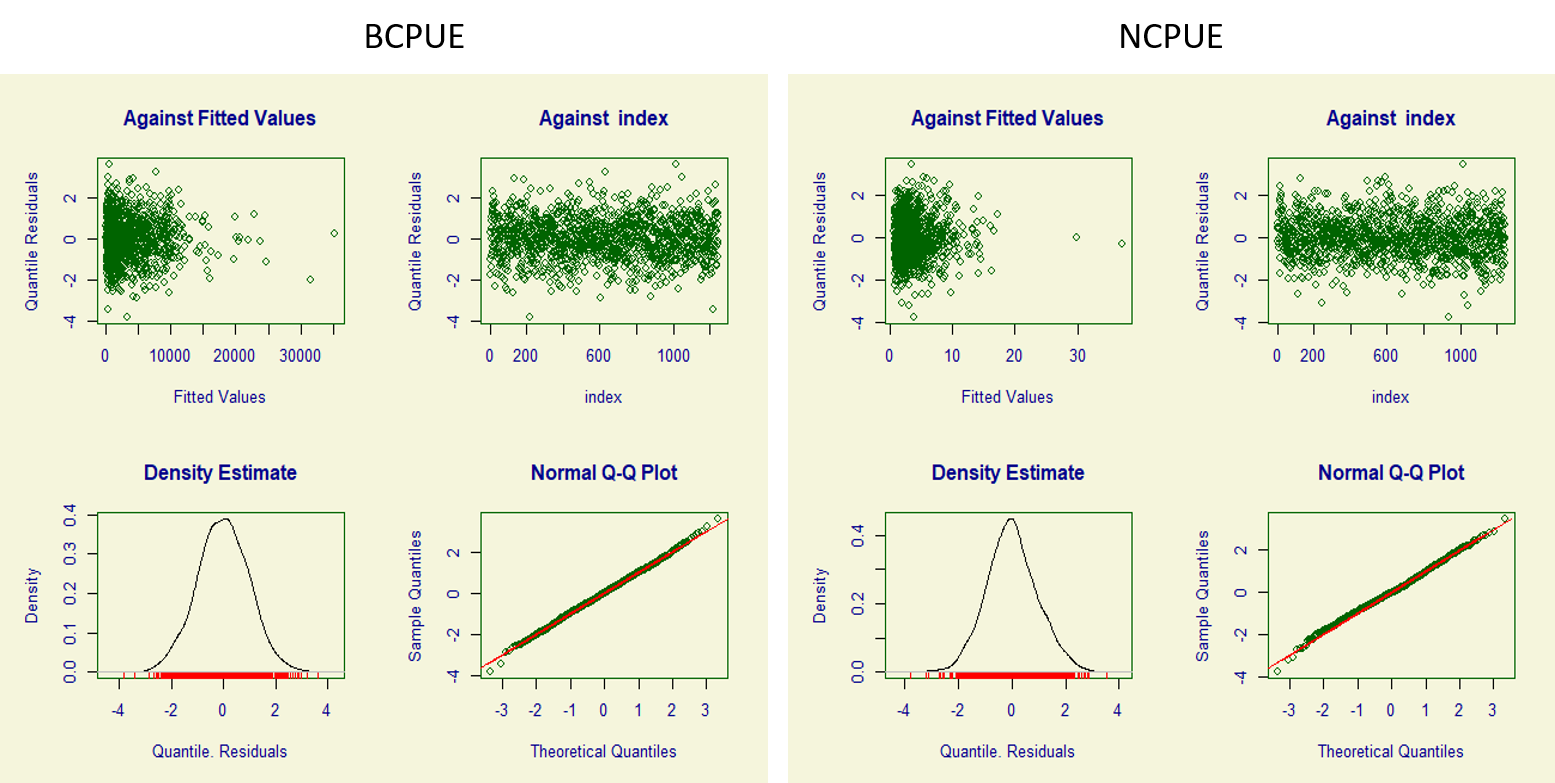


R^2^(type = "Cragg Uhler") 0.3799577 R^2^(type = "Cragg Uhler") 0.4134126

Supplementary Figure 4. **Residuals of the GAMLSS models for BCPUE and NCPUE**

**
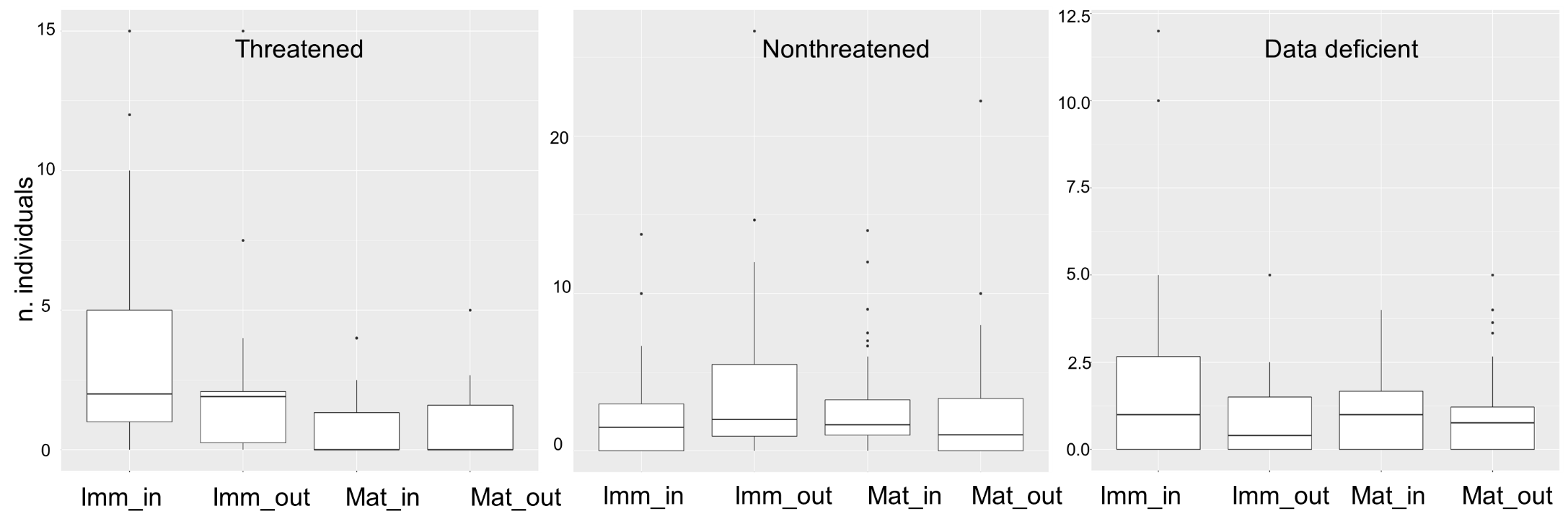
**

Supplementary Figure 5. **Immature and mature individuals of the three risk groups inside the partially protected area (in) and unprotected area (out)**

**
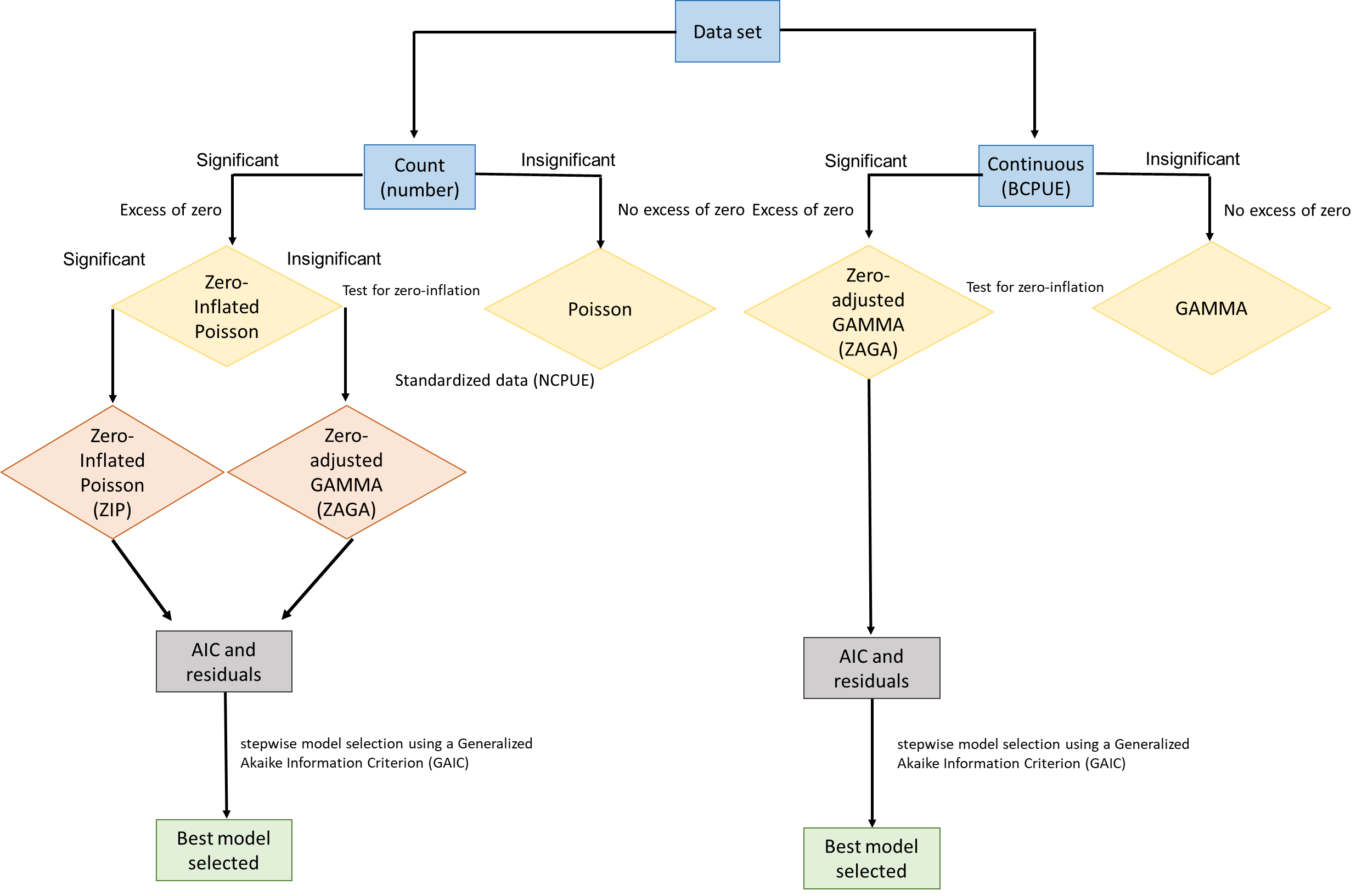
**

Supplementary Figure 6. **A flow-chart showing the procedure used for the selection of the best model**

Supplementary Table 1. **Selection of the candidate predictors considered in this study.** Studies suggesting a relationship of the variable to distribution of elasmobranchs

| Category | Explanatory variable | Abbreviation | Type | factor | Source | Previous studies |
| --- | --- | --- | --- | --- | --- | --- |
| Antrhopogenic | Protection | in.out | Categorical | Fixed | present survey | present work |
| Antrhopogenic | Human pressure | hp | Continuous | Fixed | Micheli et al 2013 | ^1^ |
| Geographic | location | loc | Categorical | Random | present survey | present work |
| Geographic | Longitude | lon | Continuous | Fixed | present survey | present work |
| Geographic | Latitude | lat | Continuous | Fixed | present survey | present work |
| Temporal | Season | time | Categorical | Fixed | present survey | present work |
| Bathymetric | Depth | depth | Continuous | Fixed | present survey | ^2–6,8,9^ |
| Bathymetric | Type of substrate | habitat | Categorical | Random | EMODnet | ^3^ |
| Environmental | Chlorophyll a | chl-a | Continuous | Fixed | Copernicus | ^3,6,9^ |
| Environmental | Sea surface salinity | sss | Continuous | Fixed | Copernicus | ^6,8,9^ |
| Environmental | Dissolved oxygen | dox | Continuous | Fixed | Copernicus | ^6^ |
| Environmental | Phosphate | pho | Continuous | Fixed | Copernicus | ^7^ |
| Environmental | Nitrate | nit | Continuous | Fixed | Copernicus | ^7^ |
| Environmental | Sea Surface Temperature | sst | Continuous | Fixed | Copernicus | ^2–10^ |

Supplementary Table 2. **Total elasmobranch’ catches.** Average (±se) Biomass and Abundance per unit of effort (BCPUE and NCPUE, respectively mean of the elasmobranch taxa identified. For each species observations from all the locations are pooled.

| IUCN’ Group | IUCN’ Category | *Species* | Total BCPUE  (g/1000m net) | Total NCPUE  (n/1000m net) |
| --- | --- | --- | --- | --- |
| High Risk (HR) | Critically Enadangered | *Dipturus batis* | 10.0±10.0 | 0.003±0.003 |
|  | Critically Enadangered | *Leucoraja fullonica* | 0.6±0.6 | 0.002±0.002 |
|  | Endangered | *Raja radula* | 52.2±10.8 | 0.10±0.01 |
|  | Endangered | *Rhinobatos rhinobatos* | 0.5±0.5 | 0.001±0.001 |
|  | Endangered | *Rostroraja alba* | 79.7±35.2 | 0.02±0.008 |
|  | Vulnerable | *Dasyatis pastinaca* | 40.4±10.7 | 0.07±0.01 |
|  | Vulnerable | *Mustelus mustelus* | 250.5±53.8 | 0.14±0.02 |
|  | Vulnerable | *Mustelus punctulatus* | 23.22±14.37 | 0.02±0.01 |
|  | Vulnerable | *Myliobatis aquila* | 50.8±17.8 | 0.03±0.01 |
| Nonthreatened (NTH) | Least Concern | *Pteroplatytrygon violacea* | 7.7±6.1 | 0.0030.001 |
|  | Least Concern | *Raja miraletus* | 4.8±2.3 | 0.04±0.01 |
|  | Least Concern | *Raja montagui* | 2.9±1.2 | 0.01±0.004 |
|  | Least Concern | *Raja polystigma* | 5.4±1.8 | 0.04±0.01 |
|  | Least Concern | *Scyliorhinus canicula* | 80.1±23.3 | 0.26±0.05 |
|  | Least Concern | *Torpedo marmorata* | 62.2±25.7 | 0.07±0.01 |
|  | Least Concern | *Torpedo torpedo* | 64.2±24.0 | 0.01±0.004 |
|  | Near Threatened | *Dipturus oxyrinchus* | 0.3±0.3 | 0.0007±0.0007 |
|  | Near Threatened | *Leucoraja naevus* | 0.8±0.3 | 0.01±0.004 |
|  | Near Threatened | *Raja asterias* | 26.2±14.3 | 0.02±0.007 |
|  | Near Threatened | *Raja brachyura* | 21.7±14.7 | 0.02±0.008 |
|  | Near Threatened | *Raja clavata* | 47.5±15.9 | 0.03±0.01 |
|  | Near Threatened | *Raja undulata* | 0.1±0.1 | 0.001±0.001 |
|  | Near Threatened | *Scyliorhinus stellaris* | 81.5±35.5 | 0.05±0.01 |
| Data Deficient (DD) | Data Deficient | *Dasyatis tortonesei* | 2.5±1.0 | 0.01 |
| N/A | N/A | *Raja* spp. | 31.0±10.14 | 0.02±0.006 |

Supplementary Table 3. **Elasmobranchs’ catches in 11 Mediterranean MPAs.** a) Total BCPUE (g./1000 meters; mean±SE) and b) total NCPUE (n/1000 meters; mean±SE) of each species in each MPA.

a)

| Species | Bonifacio | Cabo de Palos | Cap Roux | Cote Bleue | Egadi | Es Freus | Portofino | Strunjan | Telascica | Torre Guaceto | Zakynthos |
| --- | --- | --- | --- | --- | --- | --- | --- | --- | --- | --- | --- |
| *Dasyatis pastinaca* | 221±93 | / | / | / | / | 63±21 | / | / | / | 77±36 | 9±6 |
| *Dasyatis tortonesei* | / | / | / | / | / | 16±7 | / | / | 11±11 | 0.16±0.16 | / |
| *Dipturus batis* | 100±100 | / | / | / | / | / | / | / | / | / | / |
| *Dipturus oxyrinchus* | / | / | / | / | / | / | / | / | / | / | 3±3 |
| *Leucoraja fullonica* | / | / | / | / | / | 5±5 | / | / | / | / | / |
| *Leucoraja naevus* | / | / | / | / | / | 6±2 | / | / | / | / | / |
| *Mustelus mustelus* | / | 36±25 | / | / | 1328±308 | 12±12 | / | 386±174 | 1523±178 | / | / |
| *Mustelus punctulatus* | / | / | / | / | 7.4±7.4 | / | / | 183±117 | / | / | / |
| *Myliobatis aquila* | 193±111 | 17±17 | / | / | / | 234±106 | / | 0.4±0.4 | / | / | / |
| *Pteroplatytrygon violacea* | 60±60 | / | / | 17±17 | / | 3±3 | / | / | / | / | / |
| *Raja asterias* | / | 7±7 | / | 32±23 | / | 165±109 | 4±4 | 13±12 | / | 1.1±1.1 | / |
| *Raja brachyura* | / | 2.8±2.8 | / | / | / | 166±113 | / | / | / | / | / |
| *Raja clavata* | 216±110 | / | / | / | / | 201±88 | / | / | / | / | / |
| *Raja miraletus* | / | / | / | / | / | 33±17 | / | / | / | / | 4±3 |
| *Raja montagui* | / | / | 9±9 | / | / | 13±7 | / | / | / | / | 5±4 |
| *Raja polystigma* | / | 24±17 | 4±4 | 0.8±0.8 | / | 26±10 | / | / | / | / | / |
| *Raja radula* | / | 91±43 | / | / | 84±29 | 278±73 | / | / | / | / | / |
| *Raja undulata* | / | / | / | / | / | 1.4±1.03 | / | / | / | / | / |
| *Raja* spp. | / | 11±9 | / | / | / | 181±62 | 195±95 | / | / | / | / |
| *Rhinobatos rhinobatos* | / | 22±22 | / | / | / | / | / | / | / | / | / |
| *Rostroraja alba* | / | / | / | / | / | 618±274 | / | / | / | / | / |
| *Scyliorhinus canicula* | 40±23 | 6±6 | / | / | / | 576±174 | / | / | 45±36 | / | / |
| *Scyliorhinus stellaris* | 83±82 | / | 64±59 | / | / | 536±278 | / | / | / | / | / |
| *Torpedo marmorata* | / | 8±6 | / | / | 19±11 | 439±197 | / | 6±6 | / | 16±13 | / |
| *Torpedo torpedo* | 550±229 | / | / | / | / | 44±44 | / | / | / | / | / |

b)

| Species | Bonifacio | Cabo de Palos | Cap Roux | Cote Bleue | Egadi | Es Freus | Portofino | Strunjan | Telascica | Torre Guaceto | Zakynthos |
| --- | --- | --- | --- | --- | --- | --- | --- | --- | --- | --- | --- |
| *Dasyatis pastinaca* | 0.06±0.02 | / | / | / | / | 0.19±0.04 | / | / | / | 0.3±0.1 | 0.01±0.009 |
| *Dasyatis tortonesei* | / | / | / | / | / | 0.03±0.01 | / | / | 0.02±0.02 | 0.006±0.006 | / |
| *Dipturus batis* | 0.01±0.01 | / | / | / | / | / | / | / | / | / | / |
| *Dipturus oxyrinchus* | / | / | / | / | / | / | / | / | / | / | 0.006±0.006 |
| *Leucoraja fullonica* | / | / | / | / | / | 0.01±0.01 | / | / | / | / | / |
| *Leucoraja naevus* | / | / | / | / | / | 0.07±0.02 | / | / | / | / | / |
| *Mustelus mustelus* | / | 0.02±0.01 | / | / | 0.5±0.1 | 0.006±0.006 | / | 0.07±0.03 | 0.2±0.1 | / | / |
| *Mustelus punctulatus* | / | / | / | / | 0.006±0.006 | / | / | 0.05±0.02 | / | / | / |
| *Myliobatis aquila* | 0.07±0.03 | 0.01±0.01 | / | / | / | 0.06±0.02 | / | 0.01±0.01 | / | / | / |
| *Pteroplatytrygon violacea* | 0.008±0.008 | / | / | 0.01±0.01 | / | 0.006±0.006 | / | / | / | / | / |
| *Raja asterias* | / | 0.01±0.01 | / | 0.08±0.06 | / | 0.04±0.02 | 0.01±0.01 | 0.01±0.01 | / | 0.006±0.006 | / |
| *Raja brachyura* | / | 0.01±0.01 | / | / | / | 0.05±0.01 | / | / | / | / | / |
| *Raja clavata* | 0.04±0.02 | / | / | / | / | 0.1±0.03 | / | / | / | / | / |
| *Raja miraletus* | / | / | / | / | / | 0.2±0.08 | / | / | / | / | 0.01±0.009 |
| *Raja montagui* | / | / | 0.01±0.01 | / | / | 0.02±0.01 | / | / | / | / | 0.03±0.02 |
| *Raja polystigma* | / | 0.06±0.03 | 0.02±0.02 | 0.01±0.01 | / | 0.1±0.02 | / | / | / | / | / |
| *Raja radula* | / | 0.05±0.02 | / | / | 0.2±0.05 | 0.3±0.06 | / | / | / | / | / |
| *Raja undulata* | / | / | / | / | / | 0.01±0.008 | / | / | / | / | / |
| *Raja* spp. |  |  |  |  |  |  |  |  |  |  |  |
| *Rhinobatos rhinobatos* | / | 0.01±0.01 | / | / | / | / | / | / | / | / | / |
| *Rostroraja alba* | / | / | / | / | / | 0.1±0.03 | / | / | / | / | / |
| *Scyliorhinus canicula* | 0.12±0.01 | 0.01±0.01 | / | / | / | 1.2±0.2 | / | / | 0.05±0.03 | / | / |
| *Scyliorhinus stellaris* | 0.016±0.11 | / | 0.03±0.02 | / | / | 0.1±0.06 | / | / | / | / | / |
| *Torpedo marmorata* | / | 0.02±0.01 | / | / | 0.02±0.01 | 0.2±0.04 | / | 0.006±0.006 | / | 0.1±0.08 | / |
| *Torpedo torpedo* | 0.05±0.02 | / | / | / | / | / | / | / | / | / | / |

Supplementary Table 4. **Elasmobranchs size.** Average lengths (cm), respective standard-deviation (SD) and size ranges of the elasmobranch species caught in 11 Mediterranean Marine Protected Area.

| *Species* | Total | | | Bonifacio | | | Capo de Palos | | | Cap Roux | | | Cote Bleue | | | Egadi | | |
| --- | --- | --- | --- | --- | --- | --- | --- | --- | --- | --- | --- | --- | --- | --- | --- | --- | --- | --- |
|  | **n** | **Size range** | **Mean±SD** | **n** | **Size range** | **Mean±SD** | **n** | **Size range** | **Mean±SD** | **n** | **Size range** | **Mean±SD** | **n** | **Size range** | **Mean±SD** | **n** | **Size range** | **Mean±SD** |
| *Dasyatis pastinaca* | 87 | 12 -88 | 45.06±15 | 8 | 51-70 | 59.87±5.22 | - | - | - | - | - | - | - | - | - | - | - | - |
| *Dasyatis tortonesei* | 8 | 23-91 | 51.62±21.56 | - | - | - | - | - | - | - | - | - | - | - | - | - | - | - |
| *Dipturus batis* | 2 | 58-61 | 59.5±2.12 | 2 | 58-61 | 59.5±2.12 | - | - | - | - | - | - | - | - | - | - | - | - |
| *Dipturus oxyrinchus* | 1 | 62 | 62.00 | - | - | - | - | - | - | - | - | - | - | - | - | - | - | - |
| *Leucoraja fullonica* | 2 | 87-88 | 87.5±0.71 | - | - | - | - | - | - | - | - | - | - | - | - | - | - | - |
| *Leucoraja naevus* | 12 | 25-72 | 46.83±11.56 | - | - | - | - | - | - | - | - | - | - | - | - | - | - | - |
| *Mustelus mustelus* | 105 | 49-141 | 80.77±18.54 | - | - | - | 2 | 63-66 | 64.5±2.12 | - | - | - | - | - | - | 81 | 49-141 | 81.07±18.38 |
| *Mustelus punctulatus* | 9 | 47-82 | 69.33±9.73 | - | - | - | - | - | - | - | - | - | - | - | - | 1 | 75 | 75 |
| *Myliobatis aquila* | 29 | 23-105 | 64.6±23.41 | 9 | 23-69 | 53±15.81 | 1 | 52 | 52 | - | - | - | - | - | - | - | - | - |
| *Pteroplatytrygon violacea* | 3 | 55-69 | 62.33±7.02 | 1 | 55 | 55 | - | - | - | - | - | - | 1 | 63 | 63 | - | - | - |
| *Raja asterias* | 21 | 22-97 | 57.64±17.69 | 6 | 36-80 | 62.3±19.45 | 1 | 32 | 32 | - | - | - | 8 | 56-63 | 59±2.94 | - | - | - |
| *Raja brachyura* | 11 | 30-77 | 47.45±24.29 | - | - | - | 1 | 27 | 27 | - | - | - | - | - | - | - | - | - |
| *Raja clavata* | 24 | 36-84 | 66.62±12.06 | - | - | - | - | - | - | - | - | - | - | - | - | - | - | - |
| *Raja miraletus* | 42 | 18-60 | 37.19±5.57 | - | - | - | - | - | - | - | - | - | - | - | - | - | - | - |
| *Raja montagui* | 10 | 26-57 | 41.7±8.55 | - | - | - | - | - | - | 1 | 39 | 39 | - | - | - | - | - | - |
| *Raja polystigma* | 26 | 21-72 | 41.44±10.29 | - | - | - | 6 | 21-41 | 32.16±7.06 | 2 | 42-44 | 43±1.14 | 1 | 52 | 52 | - | - | - |
| *Raja radula* | 102 | 24-65 | 44.37±9.36 | - | - | - | 5 | 24-53 | 45.02±12.02 | - | - | - | - | - | - | 33 | 29-62 | 41.45±9.78 |
| *Raja undulata* | 3 | 48-58 | 52.66±5.03 | - | - | - |  |  |  | - | - | - | - | - | - | - | - | - |
| *Rhinobatos rhinobatos* | 1 | 82 | 82.00 | - | - | - | 1 | 82 | 82 | - | - | - | - | - | - | - | - | - |
| *Rostroraja alba* | 21 | 20-125 | 65.14±37.35 | 3 | 20-65 | 36.66±24.66 |  |  |  | - | - | - | - | - | - | - | - | - |
| *Scyliorhinus canicula* | 266 | 25-111 | 41.64±9.42 | 56 | 25-50 | 40.39±5.06 | 1 | 42 | 42 |  |  |  | - | - | - | - | - | - |
| *Scyliorhinus stellaris* | 36 | 15-97 | 58±22.24 | 2 | 15-92 | 53.55±54.4 | 2 | 23-20 | 21.5±2.12 | 3 | 25-65 | 39±22.53 | - | - | - | - | - | - |
| *Torpedo marmorata* | 64 | 15-57 | 32±10.23 | - | - | - | - | - | - | - | - | - | 1 | 31 | 31 | 4 | 35-48 | 43.75±5.96 |
| *Torpedo torpedo* | 7 | 49-81 | 61.14±11.38 | 7 | 49-81 | 61.14±11.37 | - | - | - | - | - | - | - | - | - | - | - | - |

| *Species* | Es Freus | | | Portofino | | | Strunjan | | | Telascica | | | Torre Guaceto | | | Zakynthos | | |
| --- | --- | --- | --- | --- | --- | --- | --- | --- | --- | --- | --- | --- | --- | --- | --- | --- | --- | --- |
|  | **n** | **Size range** | **Mean±SD** | **n** | **Size range** | **Mean±SD** | **n** | **Size range** | **Mean±SD** | **n** | **Size range** | **Mean±SD** | **n** | **Size range** | **Mean±SD** | **n** | **Size range** | **Mean±SD** |
| *Dasyatis pastinaca* | 32 | 30_74 | 52.5±11.48 | - | - | - | - | - | - | - | - | - | 45 | 12_88 | 37.28±13.87 | 2 | 38_-60 | 49±15.55 |
| *Dasyatis tortonesei* | 6 | 48_91 | 60.33±16.86 | - | - | - | - | - | - | 1 | 23 | 23 | 1 | 28 | 28 | - | - | - |
| *Dipturus batis* | - | - | - | - | - | - | - | - | - | - | - | - | - | - | - | - | - | - |
| *Dipturus oxyrinchus* | - | - | - | - | - | - | - | - | - | - | - | - | - | - | - | 1 | 62 | 62 |
| *Leucoraja fullonica* | 2 | 87_88 | 87.5±0.70 | - | - | - | - | - | - | - | - | - | - | - | - | - | - | - |
| *Leucoraja naevus* | 12 | 25_72 | 46.83±11.55 | - | - | - | - | - | - | - | - | - | - | - | - | - | - | - |
| *Mustelus mustelus* | 1 | 85 | 85 | - | - | - | 12 | 51_89 | 66.1±12.89 | 9 | 77_111 | 97.5±12.60 | - | - | - | - | - | - |
| *Mustelus punctulatus* | - | - | - | - | - | - | 8 | 47_82 | 68.62±10.15 | - | - | - | - | - | - | - | - | - |
| *Myliobatis aquila* | 17 | 41_105 | 77.23±23.43 | - | - | - | 2 | 40_42 | 41±1.41 | - | - | - | - | - | - | - | - | - |
| *Pteroplatytrygon violacea* | 1 | 69 | 69 | - | - | - | - | - | - | - | - | - | - | - | - | - | - | - |
| *Raja asterias* | 8 | 36_97 | 66.75±17.26 | 1 | 62 | 62 | 2 | 22_52 | 37±21.21 | - | - | - | 1 | 42 | 42 | - | - | - |
| *Raja brachyura* | 9 | 34_77 | 54.66±19.48 | - | - | - | - | - | - | - | - | - | 1 | 30 | 30 | - | - | - |
| *Raja clavata* | 18 | 52_84 | 68.05±8.75 | - | - | - | - | - | - | - | - | - | - | - | - | - | - | - |
| *Raja miraletus* | 40 | 18_60 | 37.35±5.54 | - | - | - | - | - | - | - | - | - | - | - | - | 2 | 29_39 | 34±7.07 |
| *Raja montagui* | 4 | 38_47 | 42.25±3.77 | - | - | - | - | - | - | - | - | - | - | - | - | 5 | 26_57 | 41.8±12.31 |
| *Raja polystigma* | 17 | 35_72 | 44.06±10.16 | - | - | - | - | - | - | - | - | - | - | - | - | - | - | - |
| *Raja radula* | 64 | 29_65 | 45.81±8.71 | - | - | - | - | - | - | - | - | - | - | - | - | - | - | - |
| *Raja undulata* | 3 | 48_58 | 52.66±5.03 | - | - | - | - | - | - | - | - | - | - | - | - | - | - | - |
| *Rhinobatos rhinobatos* | - | - | - | - | - | - | - | - | - | - | - | - | - | - | - | - | - | - |
| *Rostroraja alba* | 18 | 20_125 | 69.88±37.45 | - | - | - | - | - | - | - | - | - | - | - | - | - | - | - |
| *Scyliorhinus canicula* | 207 | 28_76 | 39.75±10.20 | - | - | - | - | - | - | 2 | 55_76 | 65.5±14.84 | - | - | - | - | - | - |
| *Scyliorhinus stellaris* | 31 | 34_97 | 60.12±20.05 | - | - | - | - | - | - | - | - | - | - | - | - | - | - | - |
| *Torpedo marmorata* | 39 | 15_57 | 35.71±8.62 | - | - | - | 1 | 37 | 37 | - | - | - | - | - | - | - | - | - |
| *Torpedo torpedo* | - | - | - | - | - | - | - | - | - | - | - | - | 17 | 15_40 | 21.60±5.69 | - | - | - |

Supplementary Table 5. **BCPUE and NCPUE data modeled with the ZAGA distribution model.** The analyses were performed with 9 of 11 locations, Cote Bleue and Cap Roux were removed from the analyses.

|  | BCPUE | | |  | NCPUE | | |
| --- | --- | --- | --- | --- | --- | --- | --- |
| Log (µ) |  |  |  | **Log (µ)** |  |  |  |
|  | Coefficient | t value | Pr(>\|t\|) |  | Coefficient | t value | Pr(>\|t\|) |
| (Intercept) | 8.697203 | 24.314 | < 2e-16*** | (Intercept) | 0.933005 | 3.897 | <0.001*** |
| cs(chl-a) | 1.779333 | 6.962 | <0.001*** | cs(chl-a) | 0.60643 | 3.703 | <0.001*** |
| in.outOutside | -0.3833 | -2.81 | <0.001*** | cs(hum.impacts) | 0.049252 | 2.621 | 0.008 ** |
| cs(depth) | 0.009535 | 2.418 | 0.01*** | in.outOutside | -0.26145 | -2.938 | 0.003 ** |
| cs(sst) | -0.06292 | -3.69 | <0.001*** | cs(depth) | 0.005347 | 2.309 | 0.021 * |
|  |  |  |  | cs(sst) | 0.004446 | 0.389 | 0.697257 |
|  |  |  |  |  |  |  |  |
| Log (σ) |  |  |  | **Log (σ)** |  |  |  |
| (Intercept) | 0.0671 | 1.756 | 0.0794 | (Intercept) | -0.38055 | -9.088 | <2e-16 *** |
|  |  |  |  |  |  |  |  |
| Logit (ν) |  |  |  | **Logit (ν)** |  |  |  |
| (Intercept) | 1.545671 | 3.672 | <0.001*** | (Intercept) | 1.580311 | 3.709 | <0.001*** |
| in.outOutside | 0.511209 | 2.958 | 0.003** | in.outOutside | 0.491953 | 2.823 | 0.004 ** |
| cs(depth) | -0.01683 | -3.948 | <0.001*** | cs(depth) | -0.01793 | -4.771 | 2.06e*** |
| cs(sst) | 0.015858 | 0.756 | 0.44958 | cs(sst) | 0.019431 | 0.918 | 0.358959 |
|  |  |  |  |  |  |  |  |
| No. of observations | 1087 |  |  | No. of observations | 1087 |  |  |
| Degrees of Freedom | 67.29044 |  |  | Degrees of Freedom | 67.29044 |  |  |
| Residual Deg. | 1179.71 |  |  | Residual Deg. | 1179.71 |  |  |
| Global Deviance: | 5476.941 |  |  | Global Deviance: | 2001.138 |  |  |
| AIC: | 5611.522 |  |  | AIC: | 2140.619 |  |  |
| SBC: | 5956.621 |  |  | SBC: | 2498.281 |  |  |

Supplementary Table 6. **Restrictions and features of fishing operation.** Summary of fishery regulations in the partially protected areas (PPAs) and unprotected areas (UPA) in each location and features of fishing operation considered in this study. m = meters; mm = millimeter; h = hour; se = standard error

|  | Restrictions | | | | | | | Features of fishing operation | | | |
| --- | --- | --- | --- | --- | --- | --- | --- | --- | --- | --- | --- |
| Location | **Zone** | **Limited entry** | **Gear restrictions** | **Time restrictions** | **Total allowable catch** | **Fish size limits** | **Territorial Rights** | **Depth (m)**  **(mean±se)** | **Mesh (mm)**  **(mean±se)** | **Soak time (h)**  **(mean±se)** | **Net.length (m)**  **(mean±se)** |
| Bonifacio | PPA |  | X | X |  |  | X | 51.71±1.8 | 39.73±1.03 | 36.83±1.66 | 434.15±10.26 |
| Bonifacio | UPA |  |  |  |  |  |  | 53.938±6.11 | 41.29±2.18 | 43.33±5.19 | 458.33±16.93 |
| Cabo de Palos | PPA | X | X | X |  | X | X | 27.54±0.9 | 33.04±2.09 | 4.6±0.57 | 340±15.27 |
| Cabo de Palos | UPA |  |  |  |  |  |  | 13.812±0.6 | 41.37±0.24 | 3.64±0.12 | 429.68±11.71 |
| Cap Roux | UPA |  |  |  |  |  |  | 64.62±6.2 | 32.43±1.02 | 25.64±1.78 | 549.36±30.93 |
| Cote Bleue | UPA |  |  |  |  |  |  | 24.46±2.0 | 32.32±0.95 | 14.64±0.77 | 1648.92±112.88 |
| Egadi | PPA | X | X | X |  |  |  | 28.31±1.19 | 30.70±0.63 | 11.18±0.37 | 1035.95±34.28 |
| Egadi | UPA |  |  |  |  |  |  | 21.33±0.8 | 28.71±0.24 | 13.87±0.15 | 701.75±21.49 |
| Es Freus | PPA | X | X | X |  | X |  | 22.5±0.9 | 42.63±1.11 | 19.13±0.40 | 806.08±63.27 |
| Es Freus | UPA |  |  |  |  |  |  | 31.49±1.5 | 43.04±0.855 | 20.32±0.53 | 767.33±34.02 |
| Portofino | PPA | X |  |  |  | X |  | 27.61±2.20 | 35.84±1.80 | 19.48±2.69 | 540.76±40 |
| Portofino | UPA |  |  |  |  |  |  | 35.13±4.54 | 30.97±1.23 | 18.9±2.45 | 682.5±56.51 |
| Strunjan | PPA |  | X | X |  |  | X | 12.33±0.37 | 26±1 | 14.93±0.30 | 209.61±5.31 |
| Strunjan | UPA |  |  |  |  |  |  | 15.88±0.49 | 23.33±0.72 | 18.75±1.22 | 355.78±21.86 |
| Telascica | PPA |  | X | X |  |  |  | 16.47±3.15 | 20.72±1.24 | 11.73±0.61 | 244.78±26.32 |
| Telascica | UPA |  |  |  |  |  |  | 16.46±3.4 | 20.35±2.24 | 8.64±1.50 | 613.57±290.68 |
| Torre Guaceto | PPA | X | X | X |  |  | X | 14.75±0.6 | 27.87±0.39 | 10.58±0.61 | 900±28.25 |
| Torre Guaceto | UPA |  |  |  |  |  |  | 18.41±1.38 | 28.82±0.47 | 12.40±0.75 | 961.17±13.39 |
| Zakynthos | PPA |  |  | X |  |  |  | 37.28±3.01 | 26.05±0.54 | 7.30±0.20 | 1011.84±39.77 |
| Zakynthos | UPA |  |  |  |  |  |  | 38.79±2.08 | 23.29±0.37 | 7.01±0.29 | 961.42±52.17 |

Supplementary Table 7. **Length at maturity (L50) of the elasmobranch species caught during the study.** L50 for males were used to estimate the proportion of mature and immature individuals as these are more conservative than L50 for females.

| *Species* | Area | L50 Males (cm) | L50 Females (cm) | Ref |
| --- | --- | --- | --- | --- |
| *Dasyatis pastinaca* | Levantine sea (Turkey) | **46** | 49 | ^11^ |
| *Dasyatis tortonesei* | Tunisia | **68** | 83 | ^11^ |
| *Dipturus batis* | North Sea | **130** | 160 | fishbase |
| *Dipturus oxyrinchus* | Aegean sea (Turkey) | **64.5** | 82.5 | ^11^ |
| *Leucoraja fullonica* | North Atlantic | **75** | NA | fishbase, iucnredlist |
| *Leucoraja naevus* | North Sea | **55** | 55 | fishbase, iucnredlist |
| *Mustelus mustelus* | Adriatic Sea, Strait of Sicily (Italy) | **108.1** | 121.2 | ^12^ |
| *Mustelus punctulatus* | Adriatic Sea, Strait of Sicily (Italy) | **91.3** | 109.9 | ^12^ |
| *Myliobatis aquila* | Mediterranean sea | **80** | 100 | www.fishbase.org |
| *Pteroplatytrygon violacea* | Mediterranean sea | **60** | 70 | www.fishbase.org |
| *Raja asterias* | Tyrrhenian sea (Italy) | **50.5** | 56.5 | ^11^ |
| *Raja brachyura* | North Sea | **78.2** | 85.6 | iucnredlist |
| *Raja clavata* | Turkey | **66.7** | 64 | ^11^ |
| *Raja miraletus* | Adriatic Sea (Italy) | **36.4** | 42.3 | ^11^ |
| *Raja montagui* | North Sea | **55** | 64 | iucnredlist |
| *Raja polystigma* | Sardinia waters | **48** | 50 | ^13^ |
| *Raja radula* | Tunisia | **47** | 56.4 | ^14^ |
| *Raja undulata* | Portugal | **73.6** | 76.2 | ^11^ |
| *Rhinobatos rhinobatos* | Tunisia | **68.9** | 78.1 | ^11^ |
| *Rostroraja alba* | Tunisia | **119** | 129 | ^14^ |
| *Scyliorhinus canicula* | Tunisia | **40** | 42 | ^11^ |
| *Scyliorhinus stellaris* | Mediterranean | **77** | 79 | Serena 2005 |
| *Torpedo marmorata* | Tyrrhenian Sea | **25.1** | 31.2 | ^11^ |
| *Torpedo torpedo* | Tyrrhenian Sea | **24.9** | 25.8 | ^11^ |

Supplementary Table 8. **VIF results among environmental variables.** Variables in bold were removed from the analyses. chl-a = chlorophyll-a; pho = phosphate; sst = sea surface temperature; nit = nitrate; dox = dissolved oxygen.

|  | depth | chl-a | salinity | pho | sst | nit | dox |
| --- | --- | --- | --- | --- | --- | --- | --- |
| depth | 1 | -0.29 | 0.12 | 0.01 | 0.33 | -0.23 | -0.08 |
| chl-a | -0.29 | 1 | 0.03 | 0.44 | -0.44 | 0.8 | 0.59 |
| salinity | 0.12 | 0.03 | 1 | 0.09 | 0.29 | 0.12 | 0.37 |
| pho | 0.01 | 0.44 | 0.09 | 1 | -0.15 | 0.63 | 0.78 |
| sst | 0.33 | -0.44 | 0.29 | -0.15 | 1 | -0.27 | -0.13 |
| nit | -0.23 | 0.8 | 0.12 | 0.63 | -0.27 | 1 | 0.79 |
| dox | -0.08 | 0.59 | 0.37 | 0.78 | -0.13 | 0.79 | 1 |
|  |  |  |  |  |  |  |  |
| VIF |  |  |  |  |  |  |  |
| human.impact | depth | chl-a | salinity | pho | sst | **nit** | **dox** |
| 1.134987 | 1.213127 | 3.317808 | 1.65399 | 3.156101 | 1.505153 | 5.080295 | 6.067717 |
| VIF |  |  |  |  |  |  |  |
| human.impact | depth | chl-a | salinity | pho | sst |  |  |
| 1.115685 | 1.185701 | 1.620334 | 1.152218 | 1.27583 | 1.482399 |  |  |

Supplementary Table 9. **Summary of Akaike’s Information Criteria results from the two models (Zero-inflated poisson-ZIP and zero-adjusted Gamma-ZAGA) describing the proportion of mature and immature of the three IUCN groups between partially protected areas (PPAs) and unprotected areas (UPAs). The model with the smallest value of the Akaike information criterion (AIC) is the best (in bold)**

| Group | family error | Model | AIC |
| --- | --- | --- | --- |
| NONTHREATENED (NTH) |  |  |  |
| Immature | ZIP | modimm_zip<-gamlss(num ~ in.out+offset(log(net), family=ZIP) | 448.19 |
| Immature | ZAGA | modimm_zag<-gamlss(den ~ in.out, family=ZAGA) | **445.50** |
| Mature | ZIP | modmat_zip<-gamlss(num ~ in.out+offset(log(net), family=ZIP) | **411.50** |
| Mature | ZAGA | modmat_zag<-gamlss(den ~ in.out, family=ZAGA) | 420.99 |
| Threatened (TH) |  |  |  |
| Immature | ZIP | modimm_zip<-gamlss(num ~ in.out+offset(log(net), family=ZIP) | **372.57** |
| Immature | ZAGA | modimm_zag<-gamlss(den ~ in.out, family=ZAGA) | 391.61 |
| Mature | ZIP | modmat_zip<-gamlss(num ~ in.out+offset(log(net), family=ZIP) | **117.28** |
| Mature | ZAGA | modmat_zag<-gamlss(den ~ in.out, family=ZAGA) | 117.54 |
| Data Deficient (DD) |  |  |  |
| Immature | ZIP | modimm_zip<-gamlss(num ~ in.out+offset(log(net), family=ZIP) | 127.44 |
| Immature | ZAGA | modimm_zag<-gamlss(den ~ in.out, family=ZAGA) | **125.22** |
| Mature | ZIP | modmat_zip<-gamlss(num ~ in.out+offset(log(net), family=ZIP) | **98.01** |
| Mature | ZAGA | modmat_zag<-gamlss(den ~ in.out, family=ZAGA) | 98.47 |

Supplementary Table 10**. Hierarchal order of the models tested to describe the CPUE and NCPUE of elasmobranchs species. The model with the smallest value of the Akaike information criterion (AIC) is the best**

| BCPUE | | NCPUE | |
| --- | --- | --- | --- |
| Model Formula (log) | **AIC** | **Model Formula (log)** | **AIC** |
| elasmo ~ 1 + cs(chl-a) + cs(hum.impacts) + cs(salinity) + in.out + season+ cs(depth) + cs(sst) + cs(pho) + re(random = ~1 \| mpa/substrate, method = REML") | 5649.61 | elasmo ~ 1 + cs(chl-a) + cs(hum.impacts) + cs(salinity) + in.out + season+ cs(depth) + cs(sst) + cs(pho) + re(random = ~1 \| mpa/substrate, method = REML") | 2175.73 |
| elasmo ~ 1 + cs(chl-a) + cs(hum.impacts) + in.out + season + cs(depth) + cs(sst) + cs(pho) + re(random = ~1 \| mpa/substrate, method = REML") | 5642.4 | elasmo ~ 1 + cs(chl-a) + cs(hum.impacts) + in.out + season + cs(depth) + cs(sst) + cs(pho) + re(random = ~1 \| mpa/substrate, method = REML") | 2168.4 |
| elasmo ~ 1 + cs(chl-a) + cs(hum.impacts) + in.out + cs(depth) + cs(sst) + cs(pho) + re(random = ~1 \| mpa/substrate, method = REML") | 5637.5 | elasmo ~ 1 + cs(chl-a) + cs(hum.impacts) + in.out + season + cs(depth) + cs(sst) + re(random = ~1 \| mpa/substrate, method = REML") | 2167.3 |
| elasmo ~ 1 + cs(chl-a) + cs(hum.impacts) + in.out + cs(depth) + cs(sst) + re(random = ~1 \| mpa/substrate, method = REML") | 5633.3 | elasmo ~ 1 + cs(chl-a) + cs(hum.impacts) + in.out + cs(depth) + cs(sst) + re(random = ~1 \| mpa/substrate, method = REML") | 2166.7 |
| elasmo ~ 1 + cs(chl-a) + in.out + cs(depth) + cs(sst) + re(random = ~1 \| mpa/substrate, method = REML") | 5631.4 |  |  |
|  |  |  |  |
| Model Formula (logit) |  | **Model Formula (logit)** |  |
| elasmo ~ 1 + cs(chl-a) + cs(hum.impacts) + cs(salinity) + in.out + season+ cs(depth) + cs(sst) + cs(pho) + re(random = ~1 \| mpa/substrate, method = REML") | 5631.42 | elasmo ~ 1 + cs(chl-a) + cs(hum.impacts) + cs(salinity) + in.out + season+ cs(depth) + cs(sst) + cs(pho) + re(random = ~1 \| mpa/substrate, method = REML") | 2166.7 |
| elasmo ~ 1 + cs(chl-a) + cs(hum.impacts) + in.out + season+ cs(depth) + cs(sst) + cs(pho) + re(random = ~1 \| mpa/substrate, method = REML") | 5626.5 | elasmo ~ 1 + cs(chl-a) + cs(hum.impacts) + in.out + season+ cs(depth) + cs(sst) + cs(pho) + re(random = ~1 \| mpa/substrate, method = REML") | 2159.1 |
| elasmo ~ 1 + cs(hum.impacts) + in.out + season+ cs(depth) + cs(sst) + cs(pho) + re(random = ~1 \| mpa/substrate, method = REML") | 5621.6 | elasmo ~ 1 + cs(chl-a) + cs(hum.impacts) + in.out + season+ cs(depth) + cs(sst) + re(random = ~1 \| mpa/substrate, method = REML") | 2152.8 |
| elasmo ~ 1 + cs(hum.impacts) + in.out + cs(depth) + cs(sst) + cs(pho) + re(random = ~1 \| mpa/substrate, method = REML") | 5617.7 | elasmo ~ 1 + cs(chl-a) + in.out + season+ cs(depth) + cs(sst) + re(random = ~1 \| mpa/substrate, method = REML") | 2148.5 |
| elasmo ~ 1 + in.out + cs(depth) + cs(sst) + cs(pho) + re(random = ~1 \| mpa/substrate, method = REML") | 5614.4 | elasmo ~ 1 + cs(chl-a) + in.out + cs(depth) + cs(sst) + re(random = ~1 \| mpa/substrate, method = REML") | 2144.4 |
| elasmo ~ 1 + in.out + cs(depth) + cs(sst) + re(random = ~1 \| mpa/substrate, method = REML") | 5611.5 | elasmo ~ 1 + in.out + cs(depth) + cs(sst) + re(random = ~1 \| mpa/substrate, method = REML") | 2140.6 |

References

1. Micheli, F., *et al.* Cumulative Human Impacts on Mediterranean and Black Sea Marine Ecosystems: Assessing Current Pressures and Opportunities. *PLoSONE* **8**(12): e79889. https://doi.org/10.1371/journal.pone.0079889

2. Tserpes, G., Maravelias, C. D., Pantazi, M. & Peristeraki, P. Distribution of relatively rare demersal elasmobranchs in the eastern Mediterranean. *Estuar. Coast. Shelf Sci.* **117**, 48–53 (2013).

3. Pennino, M. G., Muñoz, F., Conesa, D., López-Qúlez, A. & Bellido, J. M. Modeling sensitive elasmobranch habitats. *J. Sea Res.* **83**, 209–218 (2013).

4. Sguotti, C., Lynam, C. P., García-Carreras, B., Ellis, J. R. & Engelhard, G. H. Distribution of skates and sharks in the North Sea: 112 years of change. *Glob. Chang. Biol.* **22**, 2729–2743 (2016).

5. Martin, C. S. *et al.* Modelled distributions of ten demersal elasmobranchs of the eastern English Channel in relation to the environment. *J. Exp. Mar. Bio. Ecol.* **418**–**419**, 91–103 (2012).

6. Navarro, J., Cardador, L., Fernández, Á. M., Bellido, J. M. & Coll, M. Differences in the relative roles of environment, prey availability and human activity in the spatial distribution of two marine mesopredators living in highly exploited ecosystems. *J. Biogeogr.* **43**, 440–450 (2016).

7. Edgar, G. J. *et al.* Global conservation outcomes depend on marine protected areas with five key features. *Nature* **506**, 216–20 (2014).

8. Lauria, V., Gristina, M., Attrill, M. J., Fiorentino, F. & Garofalo, G. Predictive habitat suitability models to aid conservation of elasmobranch diversity in the central Mediterranean Sea. *Sci. Rep.* **5**, 13245 (2015).

9. Klippel, S., Amaral, S. & Vinhas, L. Development and evaluation of species distribution models for five endangered elasmobranchs in southwestern Atlantic. *Hydrobiologia* **779**, 11–33 (2016).

10. Follesa, M. C. *et al.* Spatial variability of chondrichthyes in the northern mediterranean. *Sci. Mar.* **83**, 81–100 (2019).

11. Tsikliras, A. C. & Stergiou, K. I. Size at maturity of Mediterranean marine fishes. *Rev. Fish Biol. Fish.* **24**, 219–268 (2014).

12. Riginella, E. *et al.* Contrasting life-history traits of two sympatric smooth-hound species: implication for vulnerability. *J. Fish Biol.* **96**, 853–857 (2020).

13. Porcu, C. *et al.* Uncommon biological patterns of a little known endemic Mediterranean skate, Raja polystigma (Risso, 1810). *Reg. Stud. Mar. Sci.* **34**, 101065 (2020).

14. Kadri, H. *et al.* Age, growth, sexual maturity and reproduction of the thornback ray, Raja clavata (L.), of the Gulf of Gabès (south-central Mediterranean Sea). *Mar. Biol. Res.* **10**, 416–425 (2014).

15. Serena, F. 2005. Field identification guide to the sharks and rays of the Mediterranean and Black Sea. FAO Species Identification Guide for Fishery Purposes, FAO, Rome.
