# Supplementary material for: Small-scale fisheries catch more threatened elasmobranchs inside partially protected areas than in unprotected areas
Source: Nat Commun. 2022 Aug 9;13:4381. doi: 10.1038/s41467-022-32035-3 (PMC9363485; doi:10.1038/s41467-022-32035-3)
Supplement: Supplementary file 3 — Reporting Summary [file 41467_2022_32035_MOESM3_ESM.pdf]

nature portfolio

Corresponding author(s): Manfredi Di Lorenzo; Antonio Di Franco

Last updated by author(s): Jun 22, 2022

Reporting Summary

Nature Portfolio wishes to improve the reproducibility of the work that we publish. This form provides structure for consistency and transparency in reporting. For further information on Nature Portfolio policies, see our Editorial Policies and the Editorial Policy Checklist.

Statistics

For all statistical analyses, confirm that the following items are present in the figure legend, table legend, main text, or Methods section.

n/a	Confirmed	
☐	☒	The exact sample size (n) for each experimental group/condition, given as a discrete number and unit of measurement
☐	☒	A statement on whether measurements were taken from distinct samples or whether the same sample was measured repeatedly
☐	☒	The statistical test(s) used AND whether they are one- or two-sided<br>*Only common tests should be described solely by name; describe more complex techniques in the Methods section.*
☐	☒	A description of all covariates tested
☐	☒	A description of any assumptions or corrections, such as tests of normality and adjustment for multiple comparisons
☐	☒	A full description of the statistical parameters including central tendency (e.g. means) or other basic estimates (e.g. regression coefficient) AND variation (e.g. standard deviation) or associated estimates of uncertainty (e.g. confidence intervals)
☐	☒	For null hypothesis testing, the test statistic (e.g. F, t, r) with confidence intervals, effect sizes, degrees of freedom and P value noted<br>*Give P values as exact values whenever suitable.*
☒	☐	For Bayesian analysis, information on the choice of priors and Markov chain Monte Carlo settings
☒	☐	For hierarchical and complex designs, identification of the appropriate level for tests and full reporting of outcomes
☒	☐	Estimates of effect sizes (e.g. Cohen's d, Pearson's r), indicating how they were calculated

Our web collection on statistics for biologists contains articles on many of the points above.

Software and code

Policy information about availability of computer code

Data collection	Data collection was performed by using nondestructive methods (photo-sampling technique at landing sites).
Data analysis	Imagej was used to estimate the total lenght of all specimen. All analyses were performed in R 3.4.1 (R Development Core Team, 2018). Density plots were built using the package "ggplot2". All functions required for model fitting and assessment were implemented using the 'gamlss' package and pseudo r-squared values for each model were obtained with function 'Rsq' using option 'Cragg Uhler'. Partial Redundacy analyses were performed using the package"vegan".

For manuscripts utilizing custom algorithms or software that are central to the research but not yet described in published literature, software must be made available to editors and reviewers. We strongly encourage code deposition in a community repository (e.g. GitHub). See the Nature Portfolio guidelines for submitting code & software for further information.

Data

Policy information about availability of data

All manuscripts must include a data availability statement. This statement should provide the following information, where applicable:

- Accession codes, unique identifiers, or web links for publicly available datasets
- A description of any restrictions on data availability
- For clinical datasets or third party data, please ensure that the statement adheres to our policy

We provide the catch data that are needed to reproduce the results in the public Figshare repository (10.6084/m9.figshare.18318878; 10.6084/m9.figshare.18318884, 10.6084/m9.figshare.18318887, 10.6084/m9.figshare.18318881). Analyses were conducted in R and the code used to produce the results is provided in R files in a public Figshare repository (10.6084/m9.figshare.18318875, 10.6084/m9.figshare.18318890, 10.6084/m9.figshare.18318893).

Field-specific reporting

Please select the one below that is the best fit for your research. If you are not sure, read the appropriate sections before making your selection.

☐ Life sciences ☐ Behavioural & social sciences ☒ Ecological, evolutionary & environmental sciences

For a reference copy of the document with all sections, see nature.com/documents/nr-reporting-summary-flat.pdf

Ecological, evolutionary & environmental sciences study design

All studies must disclose on these points even when the disclosure is negative.

Study description	We assessed elasmobranch catches of Small Scale Fisheries (SSFs) operations with fixed nets carried out in partially protected areas (PPAs) within MPAs and unprotected areas (UPA) outside MPAs at 11 locations in 6 Mediterranean countries to: (1) assess the interaction between SSFs and coastal elasmobranchs characterizing which species are fished; (2) investigate potential differences in species biomass CPUE (BCPUE, kilograms per 1000 m of net), and abundance CPUE (NCPUE, number of individuals per 1000 m of net) between PPAs vs. UPA in 9 out of 11 locations), and accounting for a set of potential covariates (e.g. chlorophyll a, sea surface temperature etc.)
Research sample	We use an empirical dataset of the elasmobranch catches from 1,257 fishing operations of Small-Scale fisheries. Each row of the data holds information on one fishing operations (including environmetal, geographical, temporal, bathymetric and anthropogenic variables) and the species. The individuals were sampled directly in the field using photo-sampling technique to obtain the size of each specimen.
Sampling strategy	In order to obtain the most comprehensive dataset possible, and considering that different fishers may have different fishing habits, we monitored catches from as many fishers as possible among those willing to take part in the assessment . A similar sampling effort was applied in all areas in order to monitor a comparable number of SSF catches. In a few cases, the relatively small size of the SSF communities and prolonged adverse meteorological conditions, especially in the winter season, contributed to a reduced number of catches monitored compared to the majority of the areas.
Data collection	Data were collected by the operator from each Marine Protected Area using photo-sampling technique at landing sites to record the SSF catches. We have chosen this method to minimize sampling time in the field and fish manipulation, thus minimising the level of disturbance for fishers. A trained operator processed the images using the image-analysis free software ImageJ. Each species has also been associated to its Mediterranean IUCN category
Timing and spatial scale	All data were collected between June-2017 to Oct-2018 across the Mediterranean Sea. Specifically, data have been collected at 11 Marine Protected Areas (sensu lato, including area established under different designations) located in 6 EU countries of the Mediterranean Sea: Bonifacio, Cap Roux, Côte Bleue (France), Portofino, Egadi and the southern Trapani coast, Torre Guaceto (Italy), Es Freus, Cabo de Palos (Spain), Telascica (Croatia), Strunjan (Slovenia) and Zakynthos island (Greece).
Data exclusions	Whenever one or more specimens were not completely visible from pictures, the catch was not retained for further analyses (36 out of 1,292 cases, i.e. 2.8% of all catches)
Reproducibility	In order to ensure reproducibility of experimental findings we developed and reported in the manuscript data collection and data analyses protocols. We developed a data collection protocol (as detailed in the manuscript) to ensure the reproducibility of data collection process. For data analyses we followed standard procedures, as reported in the manuscript, to ensure reproducibility of data analyses.
Randomization	Data were collected randomly from professional fishers at several locations. All relevant covariates were used as control: environmental (chlorophyll a, sea surface salinity, dissolved oxygen, phosphate, nitrate, sea surface temperature and habitat), geographical (location, latitude and longitude), temporal (season), bathymetric (depth) and anthropogenic (human pressure).
Blinding	Blinding was not relevant in our study, as all the elasmobranchs found in each fishing operation were analysed.

Did the study involve field work? ☒ Yes ☐ No

Field work, collection and transport

Field conditions	Fishing operations were performed following fisher' behavior. Overall, the mean temperature of the catch site ranged between 11.2 - 28.5 °C, and the mean salinity ranged between 36,9 - 39,0 g/kg, depth ranged between -2 and -190 m.
Location	The study was conducted at 11 locations (each including both MPAs and unprotected areas) across the Mediterranean Sea (between 45.560601 and 37.604748N, between -0.609459 and 20.958846E) and covering 6 countries: Telascica (Croatia), Bonifacio, Cap Roux and Côte Bleue (France), Zakynthos (Greece), Egadi Islands, Portofino and Torre Guaceto (Italy), Strunjan (Slovenia), Cabo de Palos and Freus d'Evissa I Formentera (Spain). Coordinates of each catch site are included in the relevant dataset.
Access & import/export	The import/export statement is not relevant to our study as we collected data using nondestructive methods (photo-sampling technique at landing sites). Sampling activities were authorized by each of the 11 Marine Protected Areas that have been investigated in the study.

Disturbance	No disturbance was caused by the study.

Reporting for specific materials, systems and methods

We require information from authors about some types of materials, experimental systems and methods used in many studies. Here, indicate whether each material, system or method listed is relevant to your study. If you are not sure if a list item applies to your research, read the appropriate section before selecting a response.

Materials & experimental systems

n/a	Involved in the study
☒	☐ Antibodies
☒	☐ Eukaryotic cell lines
☒	☐ Palaeontology and archaeology
☒	☐ Animals and other organisms
☒	☐ Human research participants
☒	☐ Clinical data
☒	☐ Dual use research of concern

Methods

n/a	Involved in the study
☒	☐ ChIP-seq
☒	☐ Flow cytometry
☒	☐ MRI-based neuroimaging
